# A Novel Six-Gene Signature for Prognosis Prediction in Ovarian Cancer

**DOI:** 10.3389/fgene.2020.01006

**Published:** 2020-10-15

**Authors:** Xin Pan, Xiaoxin Ma

**Affiliations:** Department of Obstetrics and Gynecology, Shengjing Hospital of China Medical University, Shenyang, China

**Keywords:** ovarian cancer, EMT, prognostic, mRNAs, survival

## Abstract

Ovarian cancer (OC) is the most malignant tumor in the female reproductive tract. Although abundant molecular biomarkers have been identified, a robust and accurate gene expression signature is still essential to assist oncologists in evaluating the prognosis of OC patients. In this study, samples from 367 patients in The Cancer Genome Atlas (TCGA) database were subjected to mRNA expression profiling. Then, we used a gene set enrichment analysis (GSEA) to screen genes correlated with epithelial–mesenchymal transition (EMT) and assess their prognostic power with a Cox proportional regression model. Six genes (TGFBI, SFRP1, COL16A1, THY1, PPIB, BGN) associated with overall survival (OS) were used to construct a risk assessment model, after which the patients were divided into high-risk and low-risk groups. The six-gene signature was an independent prognostic biomarker of OS for OC patients based on the multivariate Cox regression analysis. In addition, the six-gene model was validated with samples from the Gene Expression Omnibus (GEO) database. In summary, we established a six-gene signature relevant to the prognosis of OC, which might become a therapeutic tool with clinical applications in the future.

## Introduction

Ovarian cancer is currently the fifth leading cause of death and has become a major threat to the reproductive health of women. According to previous reports, 80% of OC patients are initially diagnosed with advanced OC (Baldwin et al., 2015; Ferlay et al., 2015), and 28% of these patients show distant metastasis (Torre et al., 2018). Although standard therapies aimed at complete resection combined with chemotherapy and neoadjuvant therapies, such as carboplatin and paclitaxel (Elies et al., 2018) have been widely used in clinical practice, the prognosis of OC is poor, and the 5-year survival rate is only 30–50% (Tarver and Cancer Facts & Figures, 2012). The traditional FIGO (2014) staging system is the primary assessment standard for estimating the treatment and outcome of OC patients. However, the heterogeneity of OC is constantly changing. Clonal expansions in histologically normal tissues occurs not only in adults, but in childhood. The extent of clone expansion may inform the malignant potential and recurrence risk, which is a new insight in tumor therapy. For heterogeneous tumor samples at different sites or different times, the sequencing results of the same patient are theoretically inconsistent. PyClone can be used to analyze which subclones are present in the different sequencing data^1^. Each ovarian tumor can be regarded as a collection of many cells with different genetic and epigenetic traits. It is obviously impossible to completely cure OC by treating with a rigid treatment model, as individuals with the same stage and who receive the same treatment experience different outcomes (Minlikeeva et al., 2017; Testa et al., 2018). Thus, the current staging system is inadequate, and it is urgent to seek more accurate indicators to identify the high-risk population.

It is time consuming and laborious to experimentally identify key genomic mutations (structural variation or number variation). However, with the rapid development of high-throughput gene microarrays and whole-genome sequencing, numerous molecular landscapes and gene variants have been discovered in many tumors (Nakagawa and Fujita, 2018; Schuh et al., 2018). Developing a convenient and effective programmatic screening method for high-risk populations is of vital importance (Song et al., 2019). Currently, a large number of databases have been built by researchers to provide access for the exploration of genomic alterations. Increasing evidence has demonstrated that molecular biomarkers contribute to the prognosis evaluation of tumors, and searches can be conducted for cancer-related somatic mutation sites from databases such as COSMIC (Bamford et al., 2004). For example, overexpression of upregulated DLL3 is an independent prognostic predictor for endometrial cancer and could be a potential and novel tumor marker for early-stage endometrial cancer (Wang et al., 2018). The angiopoietin receptor, plasma TIE2, is a tumor vascular response biomarker for VEGF that inhibitors in metastatic colorectal cancer (Jayson et al., 2018). Nevertheless, researchers found that rather than traditional single-gene biomarkers, gene signatures comprising several genes can provide strong evidence for the prognosis and survival of patients with tumors. For example, a five-gene signature (ADM, ASPM, DCBLD2, E2F7, and KRT6A) demonstrates significant power to predict patient survival in two distinct patient cohorts with pancreatic ductal adenocarcinoma and is independent of the AJCC TNM staging of this disease (Raman et al., 2018). Cross-validation of this gene signature reported a better AUC of the ROC (≥0.8) than existing pancreatic ductal adenocarcinoma (PDAC) survival signatures. Gene signatures may provide insights into the mechanism of carcinogenesis to facilitate a preferable and ideal treatment schedule. A modified PageRank algorithm was used to establish a pipeline that can help identify driver mutations among the many differentially expressed genes to produce prognostic genes. It is also helpful in the selection of key genes in other biological processes (Wang et al., 2017). Murakami et al. (2016) proposed a scoring system through TCGA and the GEO databases that could predict the tumor response to platinum-based therapies or taxane, which could be useful to develop individualized treatments for OC. A prognostic model of high-grade serous ovarian carcinoma classification named CLOVAR was developed by the TCGA specifically for OC (Verhaak et al., 2013; Boris et al., 2016). In fact, many studies about creating prognostic models via TCGA databases, gene set enrichment analysis (GSEA) and COX analysis have emerged (Huiran et al., 2019; Wang et al., 2019), and the gene signatures concluded from other studies were different than what we identify and present below.

In this study, we used GSEA, a technique superior to traditional analysis, to screen target genes because GSEA is not limited to focusing on gene expression differences in disease biomarkers and analyzing survival changes; rather, this technique only focuses on genes with statistically significant aberrant expression (Mathur et al., 2018). For GO) and KEGG pathway enrichment analysis, researchers need to provide a clear threshold for differentially expressed genes. However, the actual changes in RNA expression observed by ChIP are usually the result of multiple negative feedback loops, and the sensitivity of expression discrepancies is different among various tissues. GSEA can avoid ignoring those genes that have no significant expression difference but contribute to biological behavior, gene function, and are even related gene regulation networks. In addition, GSEA allows for the monitoring of the expression changes in gene sets rather than in individual genes, so subtle expression changes can be included to obtain more accurate results (Dean et al., 2017; Zhang et al., 2017).

In our study, we attempted to produce enriched gene sets based on molecular signatures with the prognosis of OC in different stages. To this end, we profiled specific gene sets in 367 OC patients with integrated mRNA expression datasets from the TCGA database. A total of 197 mRNAs were related to epithelial–mesenchymal transition (EMT), and a six-gene risk signature that could predict patient outcomes and identify high-risk OC populations that indicate poor prognosis was established.

## Materials and Methods

### Patient Clinical Data Source and mRNA Expression Dataset

The mRNA expression profiles and corresponding clinical information from OC patients were extracted from the TCGA^2^ data portal^3^ (Tomczak et al., 2015). These data were imputed on an Illumina HiSeq RNA-Seq platform and comprised of one OC patient in stage I, 22 in stage II, 289 in stage III, and 55 in stage IV, with matching relative to stage, age, cancer status, grade, venous invasion, and lymphatic invasion. The above information is based on high-throughput whole-genome sequencing of each tumor sample. The abovementioned data are displayed in Table 1. Both the expression profiles and clinical characteristics can be obtained publicly, so there was no need to obtain ethics committee approval.

**TABLE 1:** Clinical pathological parameters of patients with ovary cancer in this study.

Clinical pathological parameters	*N*	%	Confirmed deaths
**Stage**			
Stage I	1	0.27	1
Stage II	22	5.99	5
Stage III	289	78.74	159
Stage IV	55	14.98	34
**Cancer status**			
Tumor free	85	23.22	3
With tumor	281	76.78	196
**Grade**			
G1	1	0.27	0
G2	42	11.48	25
G3	314	85.79	167
G4	1	0.27	1
GX	8	2.186	5
**Age**			
<58	183	50	92
>58	183	50	107
**Venous invasion**			
No	40	38.83	17
Yes	63	61.17	23
**Lymphatic invasion**			
No	47	41.23	19
Yes	97	58.77	45

### Gene Set Enrichment Analysis

Single-gene analysis yields little similarity between two independent fields of patient survival in cancer. GSEA is an approach that focuses on many biological functions, chromosomal locations, or regulatory activities to interpret genome-wide expression profiles (Fevzi et al., 2005). We used GSEA^4^ to determine whether a particular gene set shows a statistically significant difference in expression. The key point of GSEA is the ES, with which each gene is endowed. The ES reflects the degree of enrichment of gene members at both ends of the sequencing list. For the analysis results, we defined the significance of gene sets based on the following parameters: |normalized enrichment score (NES)| > 1, nominal *p*-value (NOM) < 0.05, and FDR *q*-val < 0.25.

### Construction of Risk Model

Firstly, we matched the patient’s gene expression profile with the patient’s clinicopathological parameters and selected patients with relatively complete data. The patients were divided into two groups according to stage: group 1 included stage I and stage II patients, whereas group 2 included stage III and stage IV patients. We determined the enriched cell pathways in group 2 by GSEA, after which we selected the target cell pathway according to the screening conditions and sorted enriched mRNAs. Univariate Cox regression analysis (Woodward and Overall, 1975) was launched to screen for survival-related mRNAs with *p* < 0.05, and then the multivariate Cox proportional hazards regression analysis was used to analyze mRNAs related to OS. After all of the genes were divided into high-risk [hazard ratio (HR) > 1] and protective (0 < HR < 1) groups, a prognostic risk score formula was established based on a linear combination of the expression levels weighted with regression coefficients originating from the multivariate Cox regression analysis. The formula is as follows: *Risk score* = $\sum_{i=1}^{n}Exp_{i}^{*}\beta_{i}$, where n is the number of selected genes, ExPi is the expression level of gene i, and βi represents the regression coefficient of gene i.

Patients were divided into high-risk and low-risk groups according to the median patient risk score. Differential expression and heat maps were used to analyze the different expression levels of genes that constitute the risk scores in the high-risk and low-risk groups. cBioPortal provides visualization tools for research and analysis of cancer genetic data to help decipher the molecular data obtained from cancer tissue and cytology research. We used cBioPortal to identify whether the genes that make up the risk score manifested mutations.

GEPIA^5^ is a cancer data mining website mainly based on the TGCA and GTEx projects. The content that can be analyzed also covers multiple aspects: single-gene or multi-gene analysis, cancer types. Researchers select the specific tumor data according to their needs. Here, we entered the name of the single gene and obtained its expression level in different stages of OC.

### Functional Enrichment Analysis

Gene set variation analysis^6^ was performed with R according to the researchers’ needs of the OC patients’ mRNA expression profile data. In addition to the R version of the downloaded executable files, source code and documentation, various user-written software packages were also included. Downloading R (3.4.1. Windows 64-bit) required visiting the main website^7^ and selecting the CRAN to initiate download.

We identified differentially expressed genes between high- and low-risk groups through the EDGR algorithm (Robinson et al., 2010) and analyzed the cell pathways corresponding to the genes with significant differences between the high- and low-risk groups online through the DAVID^8^.

We used GO and KEGG analyses to explore the biological processes related to the differentially expressed genes. The results of the two strategies were substantial in providing both an overall and a deep understanding of the biological systems identified by GSEA in our study. A PPI network was developed to explore the relationships among these genes using the online database STRING^9^. STRING was then used to map all of the hub genes connected with each other.

### Verification of the Risk Score via Cox Regression Analysis

We classified patients according to their risk scores and other clinicopathological parameters. Then, we used univariate Cox regression analysis to screen clinical pathological parameters related to survival followed by multivariate Cox proportional hazard regression analysis to determine whether the risk score was an independent predictor of OC. Most OC patients are diagnosed with stages III–IV disease, so the number of stages I–II OC cases is limited. Kaplan–Meier survival curves and ROC curves were created to compare the accuracy of the prognostic ability of the risk score with other pathological parameters in OC. To estimate the sensitivity and specificity of the risk score model and AUC value, ROC analysis was performed using SPSS 19.0. The visual nomogram (Roman et al., 2005) was displayed in the R software mentioned above as the total points for OS for each patient.

### Validation of the Risk Prognosis Model With a GEO Dataset

Although there are more than a dozen GEO datasets for OC, we chose the GSE9891 dataset because it included all six genes with matched survival data, and the size of the dataset is considerable. We downloaded GSE9891^10^ (Tothill et al., 2008), which comprises the clinical and gene expression data of 285 OC patients from the GEO database^11^. We matched the gene expression profiles of these patients with their clinicopathological parameters. The risk scores of the OC patients were calculated, and they were divided into a high-risk group and a low-risk group according to the median risk score. The feasibility of this prognosis model was verified based on whether there was a difference in survival between the high-risk and low-risk groups.

### RNA Isolation and Quantitative Real-Time Polymerase Chain Reaction (qRT-PCR)

Twenty pairs of OC tissues and normal ovarian tissues were obtained from patients seen at the Department of Obstetrics and Gynecology, Shengjing Hospital of China Medical University, China, from 2015 to 2019. In addition, non-cancerous ovarian tissues were collected from women who underwent a hysterectomy for diseases other than cancer. All patients provided informed consent, and this study was approved by the Ethics Committee of Shengjing Hospital of China Medical University (2018PS251K). Histological diagnosis was assessed by three experienced pathologists. No patient received local or systemic treatment preoperatively. Total RNA was extracted from cancer and normal tissues with a TRIzol reagent (Takara, Dalian, China), and cDNA was generated from total RNA using a PrimeScript RT-polymerase (Takara). qRT-PCR was performed using SYBR-Green Premix (Takara) with specific PCR primers (Sangon Biotech, Co., Ltd., Shanghai, China) for the following proteins: TGFBI, F ACTCAGCCAAGACACTATTTGA, R CTTGTATGGGCATCAATTGGAG; SFRP1, F GCTCAACAAG AACTGCCAC, R CTTGTCACACTTAAGCATCTCG; COL 16A1, F GGAAGGACTCAAATTGGAACAC, R GATCTTC TTGATGGCAGACGTC; THY1, F CCAACTTCACCAGCAAA TACAA, R ACTTGACCAGTTTGTCTCTGAG; PPIB, F TTC TTCATCACGACAGTCAAGA, R TCACATCCTTCAGGGGT TTATC; and biglycan (BGN), F GAACATGAACTGCATCGAG ATG, R ATTTTGTTGTGGTCTAGGTGGA. Glyceraldehyde-3-phosphate dehydrogenase (GAPDH) was used as an internal control, and fold-changes were calculated with the 2^–ΔΔCt^ method. The qRT-PCR data are expressed as the means ± standard deviation (SD) of three independent experiments. Statistical analyses were performed with the GraphPad Prism 6.0 software (La Jolla, CA, United States). Since the data in both groups exhibited normal distribution, two-sided Student’s *t*-test or one-way analysis of variance (ANOVA) was used to ascertain differences between the two groups. A *p*-value < 0.05 was considered statistically significant.

## Results

**FIGURE 1:**
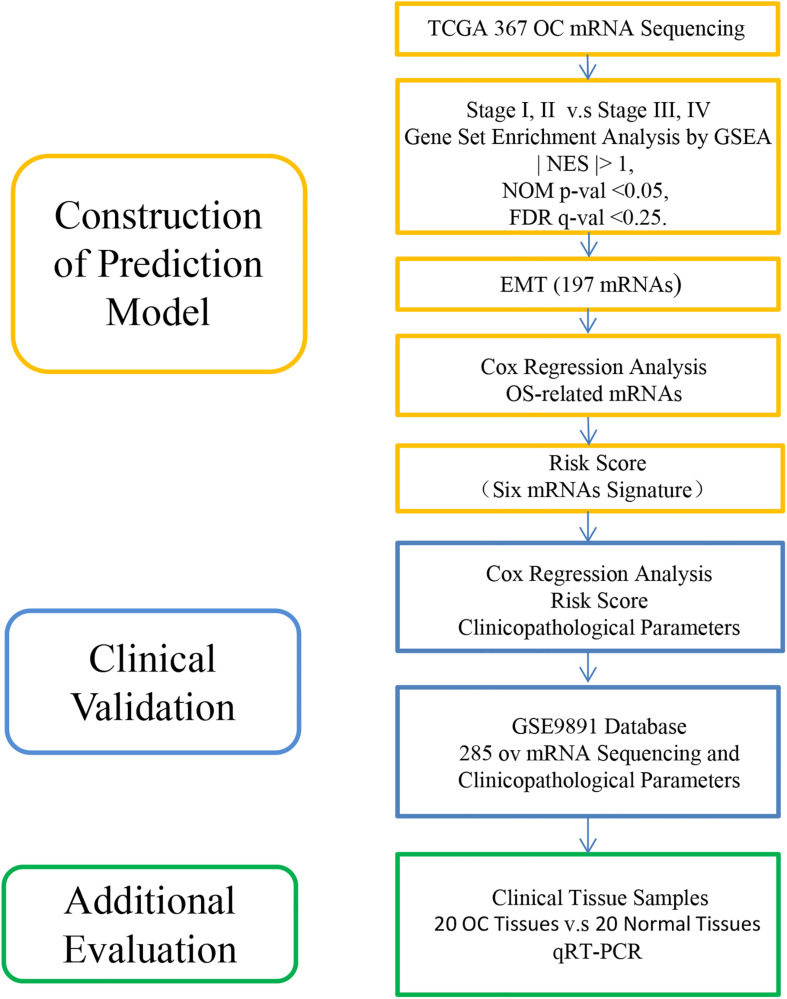
Flowchart of this study.

The flowchart of this study is shown in Figure 1. A prognostic model was established with EMT-related genes and Cox analysis, which indicated that the risk model was an independent prognostic indicator of OC.

### Gene Set Enrichment Analysis

**FIGURE 2:**
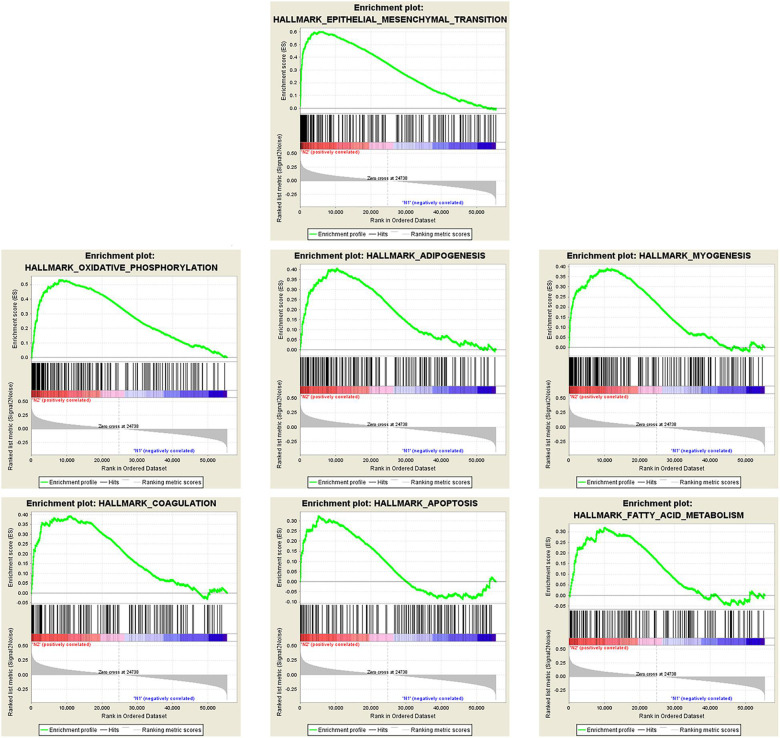
Enrichment plots of seven gene sets that were functionally enriched in OC.

There were 367 specimens (with a total of 24991 genes) from the TCGA database classified into two groups (stages I–II and stages III–IV). GSEA revealed that 24 out of the 50 hallmark biological processes were significantly enriched in the stages III–IV group. Among the 24 processes, EMT (*p* = 0), oxidative phosphorylation (*p* = 0), adipogenesis (*p* = 0), myogenesis (*p* = 0), coagulation (*p* = 0.003), apoptosis (*p* = 0.037), and fatty acid metabolism (*p* = 0.038) showed the most significant differences between the stages I–II and stages III–IV groups (Figure 2 and Table 2). A false discovery rate (FDR) < 0.25, NOM *p*-val < 0.05 and |NES| > 1were considered the cut-off criteria for each process. The top-ranking gene set was the EMT process with the highest |NES|, which includes 197 mRNAs, and was selected for further studies.

**TABLE 2:** Gene sets enriched in ovary cancer (367 samples).

GS follow link to MSigDB	Size	ES	NOM *p*-value	Rank at maximum
EPITHELIAL MESENCHYMAL TRANSITION	197	0.6	0	5691
OXIDATIVE PHOSPHORYLATION	183	0.53	0	7891
ADIPOGENESIS	190	0.41	0	10392
MYOGENESIS	199	0.39	0	10785
COAGULATION	136	0.39	0.003	10967
APOPTOSIS	159	0.32	0.037	5216
FATTY ACID METABOLISM	157	0.32	0.038	10137

### Identification of a Six-mRNA Signature Predicts Survival of OC Patients

Eighty-three of the 197 genes were selected because their expression profile was enriched in stages III–IV OC. Then, the top 19 mRNAs associated with prognosis were obtained using univariate Cox regression analysis, six of which (TGFBI, SFRP1, COL16A1, THY1, PPIB, BGN) were selected for multivariate Cox regression analysis to construct a risk assessment model. A *p*-value < 0.05 in the univariate Cox analysis was the criterion for inclusion in the multivariate Cox analysis. We designated four mRNAs as high-risk mRNAs (TGFBI, SFRP1, COL16A1, and THY1; HR > 1) and two as protective mRNAs (PPIB and BGN; 0 < HR < 1) (Table 3).

**TABLE 3:** The detailed information of six prognostic mRNAs significantly associated with overall survival in patients with ovarian cancer.

mRNA	Ensemble ID	Location	HR	B (Cox)	*p*
TGFBI	ENSG00000120708	Chr5: 136,028,988–136,063,818	1.1324	0.1243	0.0175
SFRP1	ENSG00000104332	Chr8: 41,261,962–41,309,473	1.0707	0.0683	0.0217
COL16A1	ENSG00000084636	Chr1: 31,652,263–31,704,319	1.1528	0.1422	0.0249
THY1	ENSG00000154096	Chr11: 119,417,378–119,424,985	1.0853	0.0819	0.0504
PPIB	ENSG00000166794	Chr15: 64,155,812–64,163,205	0.7530	−0.2837	0.0069
BGN	ENSG00000182492	ChrX: 153,494,980–153,509,546	0.8216	−0.1965	0.0393

A model was developed to predict prognosis according to the gene expression and regression coefficients of the six genes and is summarized as follows.

Risk score = 0.1243^∗^ TGFBI + 0.0683^∗^ SFRP1 + 0.1422^∗^ COL16A1 + 0.0819^∗^ THY1 *–* 0.2837^∗^ PPIB – 0.1965^∗^ BGN.

**FIGURE 3:**
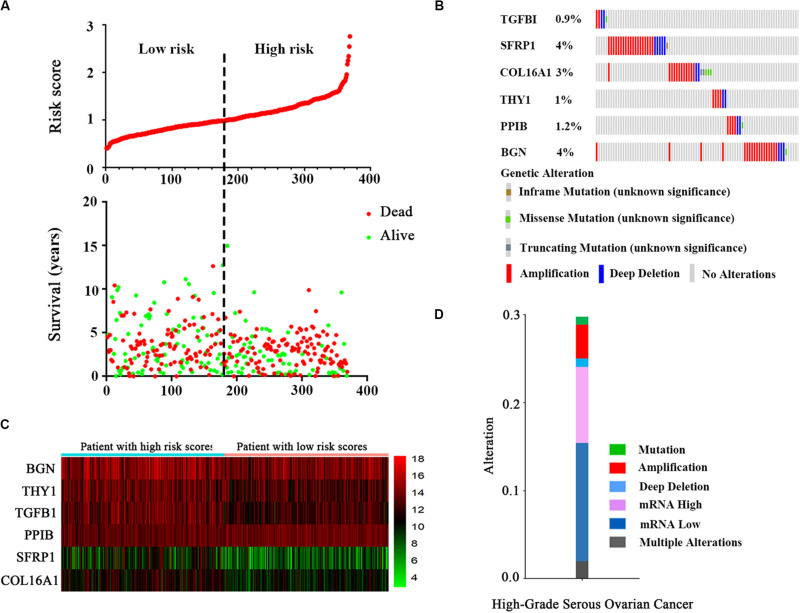
The six-mRNA signatures related to risk score predicts the OS and was associated with survival in OC patients. **(A)** mRNA risk score distribution and survival days of patients. **(B)** A heatmap of five gene expression profiles. **(C)** Selected gene alterations in the clinical samples. **(D)** Selected gene-specific alterations in the detailed cancer type.

After a risk score was calculated for every patient, the median risk score was regarded as the cut-off value, and the patients were divided into low-risk and high-risk groups (Figure 3A). The distribution of the patient relapse status is also shown in Figure 3A. The mortality increased with an increasing risk score among these patients, with a heat map (Figure 3B) indicating the expression pattern of the six mRNAs. As the risk score of the OC patients increased, the expression of the high-risk mRNAs (TGFBI, SFRP1, COL16A1, THY1) showed obvious upregulation whereas the expression of protective mRNAs (PPIB, BGN) was downregulated.

The results from the OncoPrint of cBioPortal showed a summary of the genetic alterations of the six genes in 367 OC patients. It indicated that TGFBI was altered in 0.9% patients, with two instances of amplification, two instances of deep deletions and one instance of a missense mutation (unknown significance). SFRP1, COL16A1, THY1, PPIB, and BGN showed alterations in 4, 3, 1, 1.2, and 4% of patients, respectively (Figure 3C). Similarly, the specific type of alteration in the selected genes are shown in high-grade serous OC (Figure 3D). It is clear that a loss of mRNA constituted the majority of alterations.

### Validation of the Six mRNAs for Predicting Survival by Kaplan–Meier Curves

**FIGURE 4:**
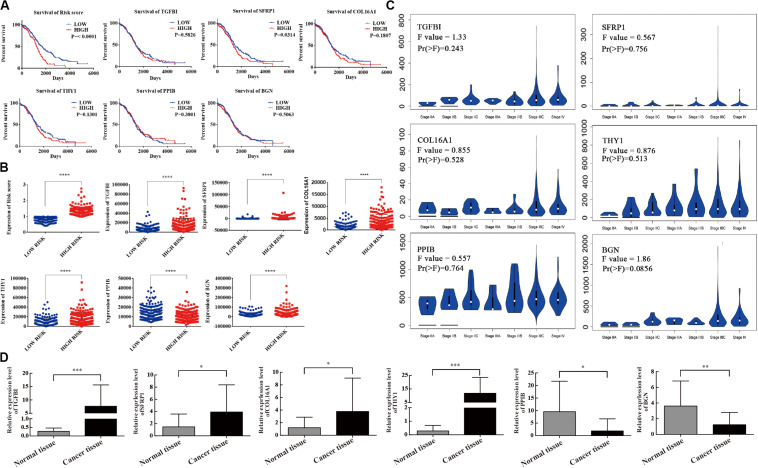
Survival validation and expression profile of the six genes grouped by high- and low-risk. **(A)** Prognostic prediction of OC by every mRNA in high- and low-risk groups. **(B)** Gene expression of the six genes in high- and low-risk groups. **(C)** Differential expression of the six genes. **(D)** The expression level of six mRNAs detected by qRT-PCR. ^∗^*P* < 0.05, ^∗∗^*P* < 0.01, ^∗∗∗^*P* < 0.001, ^****^P < 0.0001.

Kaplan–Meier curves were used to validate the prognostic value of each mRNA in predicting OC. The results showed that in addition to SFRP1 (*p* = 0.0314), the risk score was a specific indicator between the high- and low-risk groups (*p* < 0.0001). The patients in the high-risk group definitively had a shorter survival (Figure 4A). The original data of the six-mRNA expression profiles without log transformation in the high- and low-risk groups are displayed in Figure 4B. Furthermore, the mRNA expression levels of the six selected genes were tested in normal ovary tissues and OC tissues (Figure 4C). High-risk genes such as TGFBI (*p* = 0.0002), SFRP1 (*p* = 0.0344), COL16A1 (*p* = 0.0449), and THY1 (*p* < 0.0001) were overexpressed in OC tissues compared with normal tissues, while protective genes such as PPIB (*p* = 0.0128) and BGN (*p* = 0.0047) were expressed at lower levels in the OC tissues (Figure 4D).

### EMT Process Was Validated to Be Associated With the High-Risk Group of OC via GSVA Database and Functional Enrichment Analysis

**FIGURE 5:**
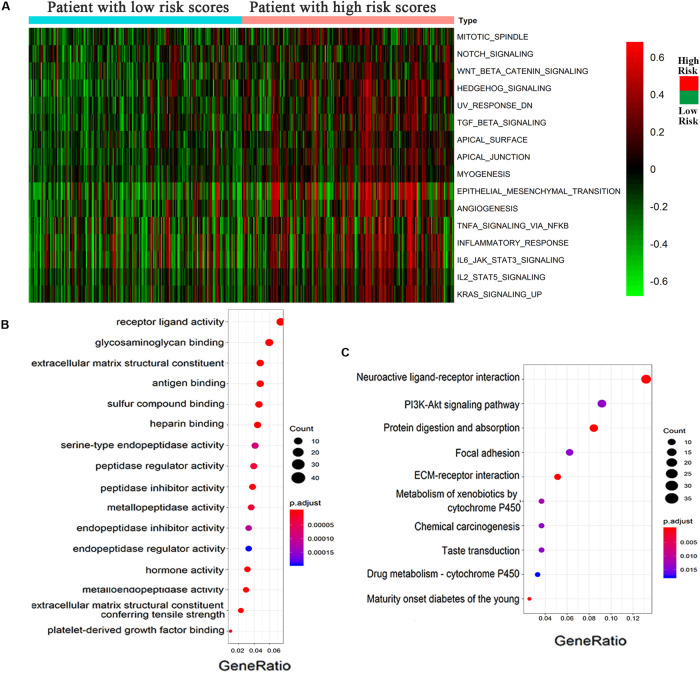
Epithelial–mesenchymal transition process was associated with the high-risk group of OC. **(A)** Heatmap displayed various biological processes associated with OC. **(B)** GO and **(C)** KEGG pathways for the functional enrichment analysis.

According to the high- and low-risk score groups obtained using samples from the GSVA database, we established a heatmap displaying various biological processes associated with OC, from which we could see that EMT was included (Figure 5A). A heatmap generated by the EDGR algorithm of the 83 most common differentially expressed genes in the high-risk and low-risk groups is shown in Supplementary Figure 1. Upregulated genes were defined as logFC > 1, and downregulated genes were defined as logFC < −1. *p* < 0.05 was considered statistically significant. The prognostic signaling pathways were evaluated by KEGG and GO pathways, from which we concluded that the genes related to prognosis were enriched in ligand receptor activity. Figures 5B,C display the GO and KEGG pathway enrichment plots, respectively, for OC. There were some differentially expressed cancer-related biological processes, such as ligand receptor activity and the PI3K-Akt signaling pathway, between the high-risk and low-risk groups, which indicated that the risk score was of great relevance to the tumorigenesis or development of OC.

### The Risk Score, Stage, and Cancer Status Are Independent Prognostic Indicators of OC

Univariate and multivariate analyses were carried out together to compare the prognostic strength of the risk score and other common clinicopathological parameters (Table 4). The results with the GSVA dataset indicated that risk score, stage, and cancer status were independent prognostic indicators since their *p*-values were < 0.05 not only in the univariate but also in the multivariate analysis. Importantly, the cancer status was the most obvious clinical parameter related to mortality for OC patients in the high-risk group, who were 8.837 times more likely to die than those in the low-risk score group. Risk score was the next most relevant factor, indicating a 2.742-fold increased likelihood of death for OC patients in the high-risk score group compared to those in the low-risk group.

**TABLE 4:** Univariable and multivariable analyses for each clinical feature.

Clinical feature	Number	Univariate analysis			Multivariate analysis		
		HR	95%CI of HR	*p*-Value	HR	95%CI of HR	*p*-Value
Risk score	367	2.742	1.855–4.053	<0.0001	1.983	1.263–3.112	0.0029
Age	367	1.023	1.010–1.035	0.0003	8.241	1.445–15.279	0.0044
Cancer Status	318	8.837	4.787–16.315	<0.0001	0.421	0.300–0.594	<0.0001
Grade	366	1.214	0.811–1.818	0.3457			
Stage	367	2.113	0.937–4.763	0.0711			
Venous invasion	102	1.026	0.611–1.722	0.924			
Lymphatic invasion	147	1.447	0.847–2.472	0.176			

### Validation of the Six-mRNA Signature for Predicting Survival by Kaplan–Meier Curves

**FIGURE 6:**
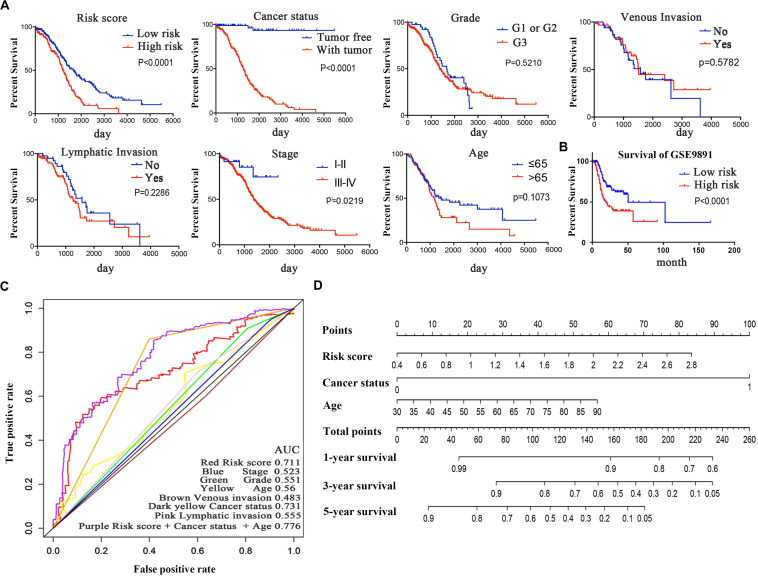
Kaplan–Meier survival analysis for the patients with OC in the TCGA dataset. **(A)** The Kaplan–Meier curve for patients divided into high-risk and low-risk groups and different clinical features that predict patient survival. **(B)** GEO validation of the signature. **(C)** ROC curves of each parameter and the combination of independent prognostic risk factors of the 5-year OS prediction. **(D)** Nomogram of total points for OS for each patient.

Kaplan–Meier curves and the log-rank method were used to validate the prognostic ability of clinical parameters (risk score, stage, age, cancer status, grade, venous invasion, and lymphatic invasion) to predict survival in OC. The results indicated that patients with high-risk scores had poor prognoses. Patients with stages III–IV disease or tumor status were at a higher risk of poor prognosis than were patients with stages I–II disease (Figure 6A). Furthermore, we performed a data stratification analysis on the entire cohort, and 367 patients were stratified based on their clinical parameters. According to the results above, patients with tumors, patients in stages III–IV or grade 3, and patients older than 65 years old were more likely to have a shorter survival (Supplementary Figure 2A). Kaplan–Meier curves were established to validate the prognostic value of the risk score to predict survival in OC in an independent GEO cohort (GSE9891). As we stated above, every patient was assigned a risk score, with patients from the GEO database divided into low-risk and high-risk groups based on the median risk score value as the cut-off criterion. The distribution of the patient relapse status is shown in Supplementary Figure 2D. It was apparent that patients with high-risk scores had poor prognoses (*p* < 0.0001), which was consistent with the result observed in Figure 6B. Kaplan–Meier curves were also used to validate the prognostic value of the risk score in predicting colon cancer (Supplementary Figure 2B) and hepatocellular cancer (Supplementary Figure 2C). The results showed that the risk score was not a significant indicator between the high- and low-risk groups (*p* = 0.1158 and *p* = 0.3675), which indicated the specificity of the risk score model to OC.

The time-dependent ROC curve of each parameter demonstrated the sensitivity and specificity of the 5-year OS prediction (Figure 6C). We identified that risk score, age, and cancer status were independent risk factors, with AUCs of 0.711, 0.56, and 0.731, respectively. In addition, we integrated these three independent risk factors into a larger model, and its AUC increased to 0.776. The AUC of the ROC curve verified the accuracy of our prognostic model. As shown in Figure 6D, each patient can be assigned point values according to the risk score, age, and cancer status in this nomogram, with the total score reflecting OS. The model is more accurate in assessing patient outcomes when the risk score, age, and cancer status are combined.

Protein–protein interaction networks obtained from the STRING database and visualized with the Cytoscape software helped us to identify the hub genes among the genes related to prognosis. The core genes (degrees ≥ 15) (Supplementary Figure 3B) were further submitted for PPI network analysis, which indicated that TGFBI, SFRP1, and COL16A1 were clearly at the center of the network (Supplementary Figure 3A).

## Discussion

In clinical practice, although two patients with the same clinical and pathological parameters received the same treatment, their outcomes were sometimes drastically different. Robust biomarkers are emerging to stratify high-risk groups among these patients. For example, the expression level of miR-205 was an independent variable selected to predict the events of progressive disease (Cañueto et al., 2017). Soluble VEGFR-2 level could predict the malignancy of ovarian neoplasms and poor prognosis in epithelial OC (Sallinen et al., 2014). The lncHIFCAR-mediated mechanism for HIF-1 activation in oral carcinoma is of great value in determining prognosis and developing a potential therapeutic strategy (Bolha et al., 2017; Shih et al., 2017). Nevertheless, increasing studies support the fact that gene regulation in biological processes is complex, with multiple genes interacting with each other to form a network. Therefore, the expression status of a single gene is insufficient to predict the prognosis of these patients (Qiu et al., 2017; Chen et al., 2018).

Epithelial–mesenchymal transition was initially studied as one of the five characteristics to differentiate benign and malignant tumors. One group proposed that an accumulation of a set of four specific cancer mutations results in the malignant transformation of normal cells (Koten and Otter, 1991; Koten et al., 1993). The EMT process has been well-studied for almost 30 years, and there have also been arguments about its mechanism as it relates to cancer (Jolly et al., 2017). In EMT, cancer cells lose their epithelial features and acquire more invasive characteristics. Activation of EMT elicits changes in multiple fundamental aspects of cellular physiology, such as wound healing, tissue fibrosis, and drug resistance, and has become a hot topic in studying carcinoma progression (Nieto et al., 2016; Shibue and Weinberg, 2017). As previously described, EMT gives rise to a variety of intermediate cell states functioning as CSCs between the epithelial and mesenchymal states (Anushka and Robert, 2019). EMT might control the expression of immune checkpoint inhibitors and promote immune evasion in non-small-cell lung carcinoma (Asgarova et al., 2018). miR-34a acts on SNAIL to regulate EMT in breast cancer and lung cancer cells (He et al., 2017). The EMT markers currently used are not consistently related to poor prognosis because the cellular context is complex.

The wide application of genomic profiling is gradually becoming the standard in clinical oncology and will undoubtedly accelerate progress in diagnosing and treating cancer (Ocak et al., 2009; Wheeler and Wang, 2013; Ouellet et al., 2019). The age of big data has arrived, and the endless screening of hub molecules has emerged. For example, researchers presented a data-interlinked platform called BIOOPENER, which enabled the query of different types of mutations and genomic alterations that may contribute to molecular and clinical insights in cancer; this approach resulted in the discovery of three pathways that potentially cause promoter changes in gynecological cancers (Jha et al., 2017). Gene signatures containing several genes are superior to individual biomarkers in predicting OC prognosis and survival and can be applied widely (Shahid et al., 2016; Liu et al., 2018; Qiao et al., 2019).

In the present study, we obtained mRNA data from 367 patients from the TCGA database, and vital bioinformatics analyses, such as GSEA, were used to reveal that EMT was potentially the most affected pathway in OC with *p* < 0.05. We then screened the 197 mRNAs related to EMT signaling and found that 83 were enriched in OC patients with stages III and IV disease. To narrow the field and improve the predictive efficiency, univariate and multivariate Cox regression analyses were performed, and six mRNAs, namely, TGFBI, SFRP1, COL16A1, THY1, PPIB, and BGN, were found to be associated with the prognosis of OC patients and subsequently combined to construct a six-gene signature model. TGFBI, SFRP1, COL16A1, and THY1 were validated to be high-risk genes, while PPIB and BGN were low-risk genes. The expression level of these genes as detected by qRT-PCR confirmed that the high-risk genes were overexpressed in OC tissues and that the protective genes were expressed at lower levels. We were able to distinguish low-risk and high-risk samples using the content of these six-mRNA signatures with relatively high prognostic accuracy. The prognosis-related biological processes and hub genes were further probed based on different risk groups. Among these mRNAs, TGFBI was investigated as a highly induced transcript during EMT in the non-small-cell lung cancer cell line A549, acting as a competing endogenous RNA (ceRNA) (Qi et al., 2015) for miR-21 to modulate EMT, which indicated that the TGFBI 3′ UTR containing the miR-21 binding site could reduce miR-21 expression and mitigate its biological function (Liu et al., 2019). SFRP1 exhibited a tumor-promoting function by selectively activating TGFβ signaling in gastric cancer cells and thus activating EMT progression (Peng et al., 2019). COL16A1 was found to be one of eight genes in a signature that predicts survival in OC and was validated to be highly expressed in cancer tissues compared with normal tissues (Wang and Li, 2020). THY1 is more highly expressed in ovarian CSCs than in non-CSCs, and high THY1 expression in patients with serous OC indicates poorer outcomes. THY1 promotes proliferation and self-renewal in OC (Elizabeth et al., 2019). BGN was indicated to be an endogenous inhibitor of bladder cancer cell proliferation by antiproliferative tyrosine kinase inhibitors. BGN is related to favorable prognosis (Christian et al., 2013). However, we also discovered that SFRP1 is thought to be a tumor suppressor that is epigenetically silenced by DNA methylation (Rashidah et al., 2020). Given all these data, what should we think about the contradictory function of a single gene? As far as we know, many genes have “dual roles” in different cancers, even in different stages of one cancer type. For example, TGF-beta inhibits the proliferation of carcinomas in the early stages of breast cancer but promotes tumor growth and metastasis in later stages of cancer (Aesun et al., 2005). In esophageal cancer cells, Nrf2 promotes cell proliferation via metabolic reprogramming and ROS detoxification (Yuki et al., 2017). By contrast, low levels of Nrf2 expression were correlated with poor survival in patients with melanoma (*p* = 0.0341), kidney cancer (*p* = 0.0203), and prostate cancer (*p* = 0.00279) (Juan et al., 2014). Therefore, the role and function of genes in cancer are complex and multifactorial; they may depend on the tumor microenvironment, targets of genes, or tumor stage, all of which need to be studied in the future.

We also investigated the prognostic value of the signature and compared it with that of other clinicopathologic parameters and different subgroups. Based on Cox analysis, the signature was able to strongly predict risk score, cancer status, and age in OC patients. It is generally considered scientific to represent tumor prognosis with 5-year OS rates. It is obvious that 5-year OS was significantly different between the high-risk and low-risk groups, revealing that the signature is a prospective independent marker of prognosis. Actually, when assessed as a ROC curve, the combination of the six-gene signature with cancer status and age exhibited a more powerful prediction for OS than did the parameters individually, indicating that comprehensive consideration of these three factors may be a promising independent tool for OC. Finally, the GEO cohort was examined to confirm the predictive capacity of this signature, and the survival result was consistent with that obtained from the original dataset. In fact, OC metastasis to the liver, colon, and other important visceral tissues is common. However, the risk score of the six-gene signature was not significant in primary liver or colon cancer, which indicated that the risk score was specific to OC prognosis. To our knowledge, this study is the first to create a prognostic gene signature in OC.

Despite the novel findings proposed by our study of candidates for OC prognosis, there are still limitations that require further investigation. This is a retrospective study, and the results would be more convincing with a larger sample size. In conclusion, the six signature genes TGFBI, SFRP1, COL16A1, THY1, PPIB, and BGN might be potential biomarkers for predicting the prognosis of OC patients. To some degree, our study might provide some clues for further investigation into the biological processes, clinical diagnosis, and therapeutic strategies of OC relating to these genes.

## Data Availability Statement

The mRNA expression and corresponding clinical information for OC patients were extracted from the TCGA data portal (https://cancergenome.nih.gov/). GEO cohort (GSE9891) was download from the GEO da (http://www.ncbi.nlm.nih.gov/gds).

## Ethics Statement

The studies involving human participants were reviewed and approved by Shengjing Hospital of China Medical University (2018PS251K). The patients/participants provided their written informed consent to participate in this study.

## Author Contributions

XP finished the experiment, analyzed the data, drafted the manuscript, and prepared all figures and tables. XM designed the study, drafted and revised the manuscript. Both authors read and approved the final manuscript.

## Conflict of Interest

The authors declare that the research was conducted in the absence of any commercial or financial relationships that could be construed as a potential conflict of interest.

## Footnotes

1 https://github.com/aroth85/pyclone

2 https://tcga-data.nci.nih.gov/tcga/

3 https://cancergenome.nih.gov/

4 http://www.broadinstitute.org/gsea/index.jsp

5 http://gepia.cancer-pku.cn/index.html

6 https://bioconductor.org/packages/release/bioc/html/GSVA.html

7 https://www.r-project.org/

8 https://david.ncifcrf.gov/

9 https://string-db.org/

10 https://www.ncbi.nlm.nih.gov/geo/query/acc.cgi?acc=GSE9891

11 http://www.ncbi.nlm.nih.gov/gds

## Supplementary Material

Supplementary Figure 1 Heatmap of 83 common differentially expressed genes in the good and poor prognosis groups.

Supplementary Figure 2 **(A)** Kaplan–Meier curves for prognostic value of risk-score signature for the patients divided by each clinical feature. **(B,C)** Prognostic prediction of OC in colon cancer and hepatocellular cancer. **(D)** Distribution of OC patients with risk scores.

Supplementary Figure 3 **(A)** PPI network analysis of prognosis-related genes. **(B)** Quantity of the genes correlated with the hub genes.

## Abbreviations

ADM: adrenomedullin
AJCC: American Joint Committee on Cancer
ASPM: abnormal spindle homolog, microcephaly associated
AUC: area under curve
BGN: biglycan Gene
ceRNA: competing endogenous RNAs
ChIP: chromatin immunoprecipitation
COL16A1: collagen type XVI alpha 1 chain
CRAN: China Video Station
CSCs: cancer stem cells
DCBLD2: discoidin, CUB and LCCL domain containing 2
DAVID: Database for Annotation, Visualization and Integrated Discovery
DLL3: delta-like protein 3
E2F7: E2F transcription factor 7
EMT: epithelial-to-mesenchymal transition
ES: enrichment score
GEO: Gene Expression Omnibus
GO: Gene Ontology
GSEA: gene set enrichment analysis
GSVA: gene set variation analysis
HR: hazard ratio
KEGG: Kyoto Encyclopedia of Genes and Genomes
KRT6A: keratin 6A
Nrf2: nuclear factor erythroid 2-related factor 2
OC: ovarian cancer
OS: overall survival
PDAC: pancreatic ductal adenocarcinoma
PPI: protein–protein interaction
PPIB: peptidylprolyl isomerase B
qRT-PCR: RNA isolation and quantitative real-time polymerase chain reaction
ROC: receiver operating characteristic
ROS: reactive oxygen species
SFRP1: secreted frizzled related protein 1
TCGA: The Cancer Genome Atlas
TGFBI: transforming growth factor beta induced
TGF-beta: transforming growth factor-beta
THY1: thy-1 cell surface antigen
TIE2: tyrosine kinase receptor
VEGF: vascular endothelial growth factor.
