# Supplementary figures and images for: A Novel Six-Gene Signature for Prognosis Prediction in Ovarian Cancer
Source: Front Genet. 2020 Oct 15;11:1006. doi: 10.3389/fgene.2020.01006 (PMC7593580; doi:10.3389/fgene.2020.01006)

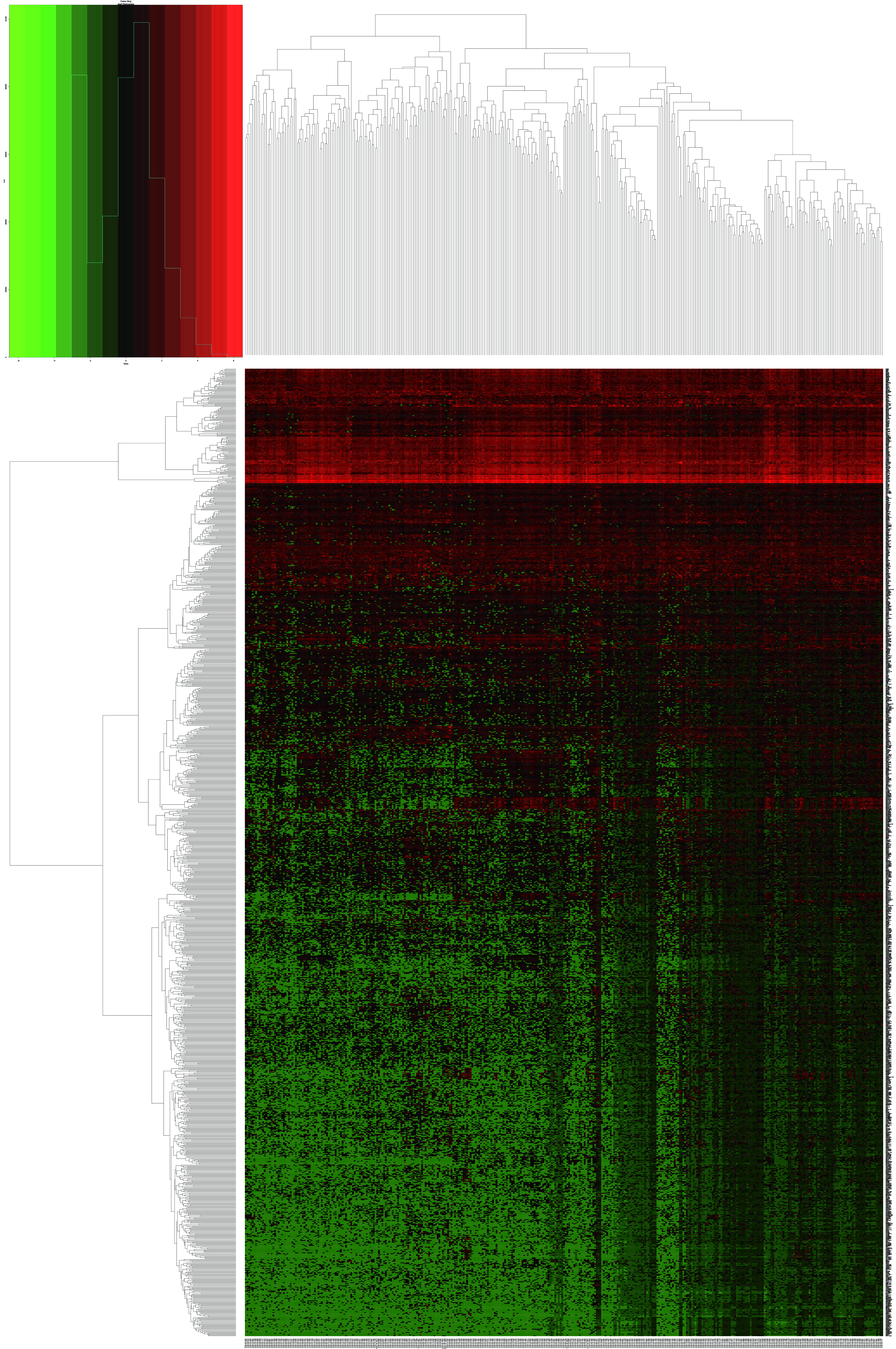

Supplement: Supplementary Figure 1 — Heatmap of 83 common differentially expressed genes in the good and poor prognosis groups. [file Image_1.TIF]

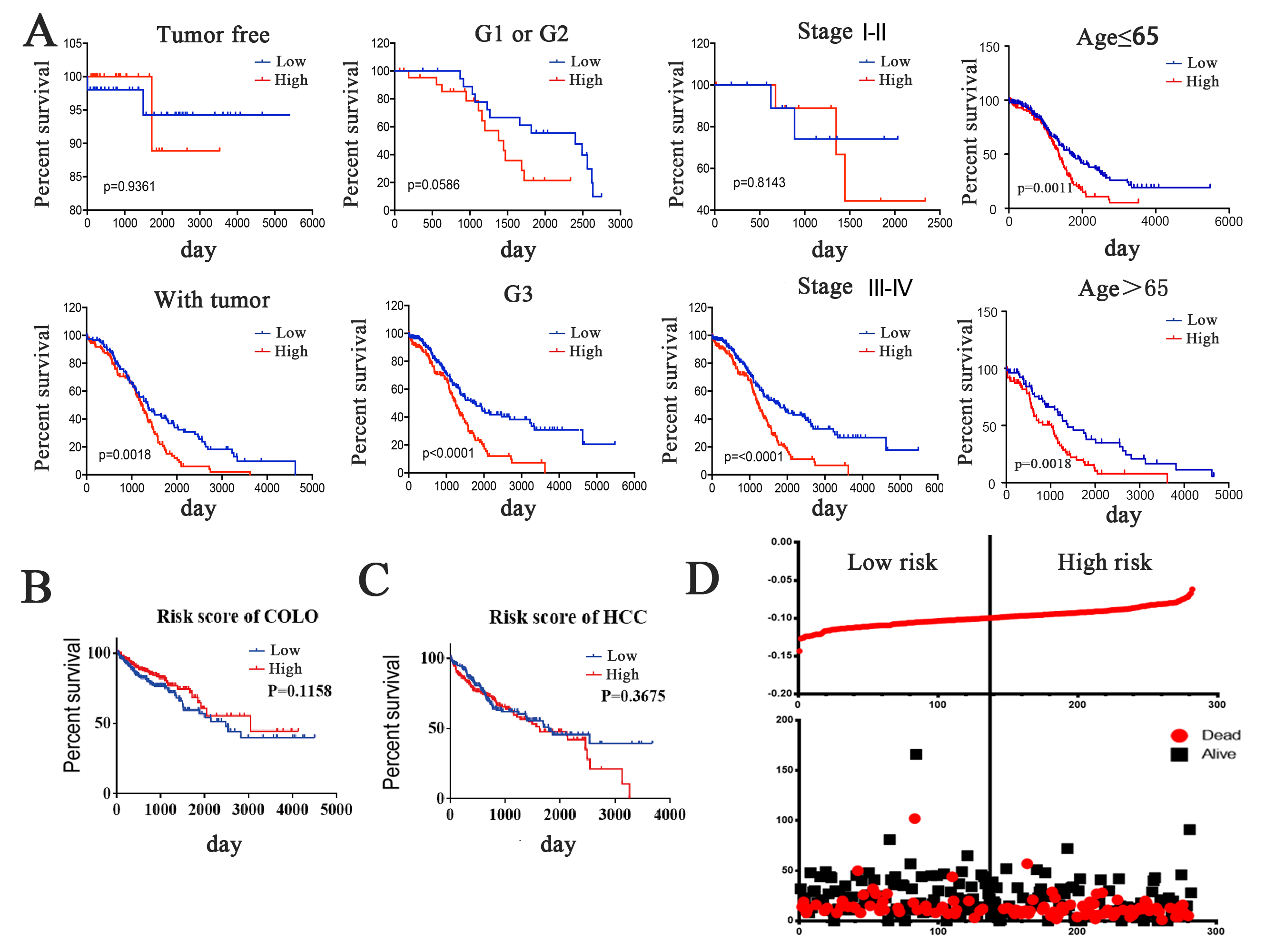

Supplement: Supplementary Figure 2 — (A) Kaplan–Meier curves for prognostic value of risk-score signature for the patients divided by each clinical feature. (B,C) Prognostic prediction of OC in colon cancer and hepatocellular cancer. (D) Distribution of OC patients with risk scores. [file Image_2.TIF]

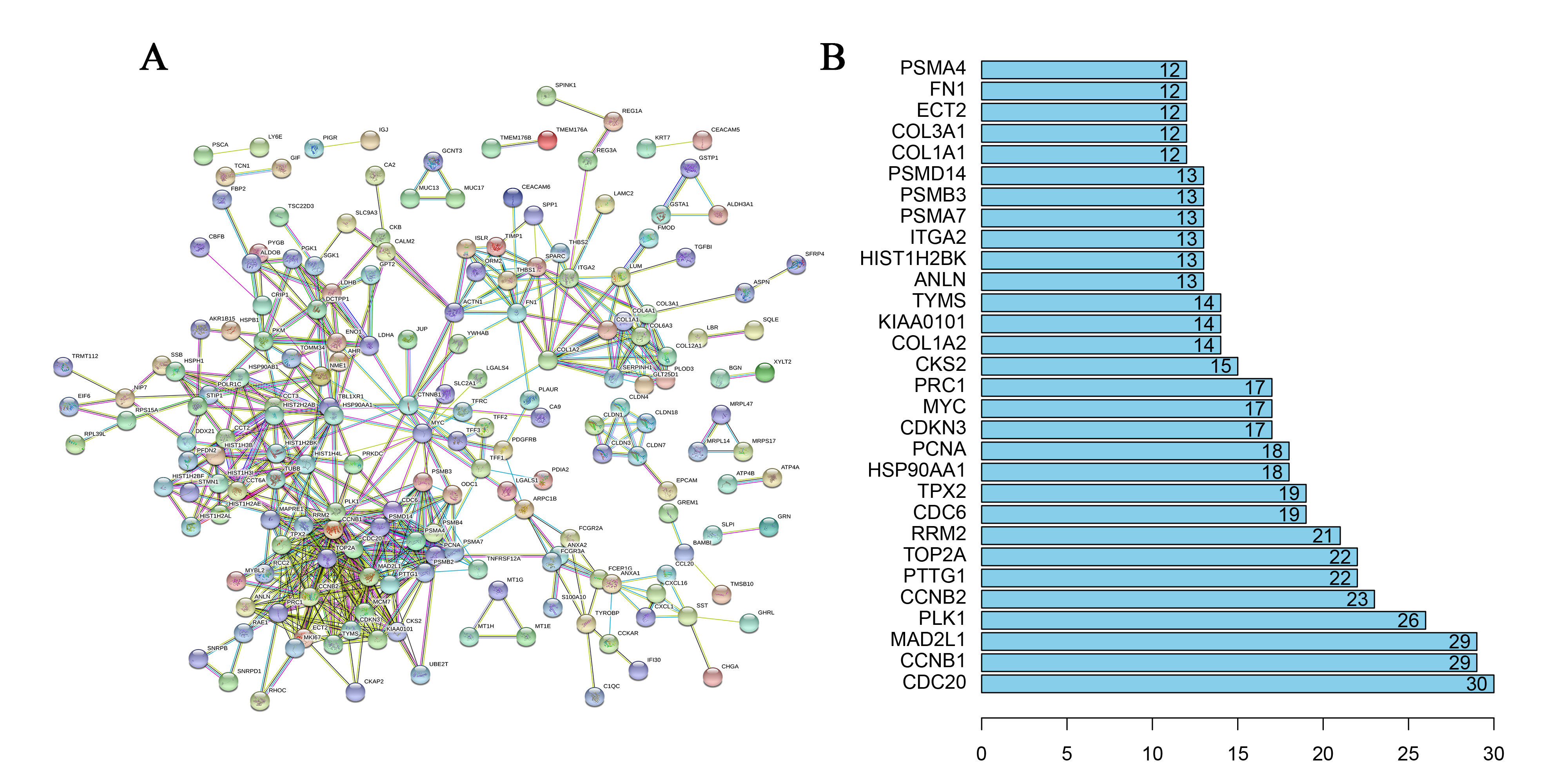

Supplement: Supplementary Figure 3 — (A) PPI network analysis of prognosis-related genes. (B) Quantity of the genes correlated with the hub genes. [file Image_3.TIF]
